# Preliminary Studies on Membrane Filtration for the Production of Potable Water: A Case of Tshaanda Rural Village in South Africa

**DOI:** 10.1371/journal.pone.0105057

**Published:** 2014-08-21

**Authors:** Gomotsegang F. Molelekwa, Murembiwa S. Mukhola, Bart Van der Bruggen, Patricia Luis

**Affiliations:** 1 ProcESS, Department of Chemical Engineering, KU Leuven, Leuven, Belgium; 2 Department of Environmental Health, Tshwane University of Technology, Pretoria, South Africa; 3 Materials and Process Engineering (iMMC-IMAP), Université Catholique de Louvain, Louvain-la-Neuve, Belgium; Dowling College, United States of America

## Abstract

Ultrafiltration (UF) systems have been used globally for treating water from resources including rivers, reservoirs, and lakes for the production of potable water in the past decade. UF membranes with a pore size of between 0.1 and 0.01 micrometres provide an effective barrier for bacteria, viruses, suspended particles, and colloids. The use of UF membrane technology in treating groundwater for the supply of potable water in the impoverished and rural village, Tshaanda (i.e., the study area) is demonstrated. The technical and administrative processes that are critical for the successful installation of the pilot plant were developed. Given the rural nature of Tshaanda, the cultural and traditional protocols were observed. Preliminary results of the water quality of untreated water and the permeate are presented. *Escherichia coli* in the untreated water during the dry season (i.e., June and July) was 2 cfu/100 ml and was <1 cfu/100 ml (undetected) following UF, which complied with the WHO and South African National Standards and Guidelines of <1 cfu/100 ml. During the wet/rainy season (February) total coliform was unacceptably high (>2419.2 cfu/100 ml) before UF. Following UF, it dramatically reduced to acceptable level (7 cfu/100 ml) which is within the WHO recommended level of <10 cfu/100 ml. Additionally, during the wet/rainy season *E. coli* and enterococci were unacceptably high (40.4 cfu/100 ml and 73.3 cfu/100 ml, respectively) before UF but were completely removed following UF, which are within the WHO and SANS recommended limit. The values for electrical conductivity (EC) and turbidity were constantly within the WHO recommended limits of 300 µS/cm corrected at 25°C and <5 NTU, respectively, before and after UF, during dry season and wet season. This suggests that there is no need for pre-treatment of the water for suspended particles and colloids. Considering these data, it can be concluded that the water is suitable for human consumption, following UF.

## Introduction

Water is a precious and valuable resource that plays a critical role in the economic and social growth of any population. Most countries around the world are still struggling to meet the demand for potable water. This is evident by the uneven distribution of and accessibility to potable water between developed and developing countries as well as between urban and rural communities [1].

About 780 million people do not have access to improved drinking water sources and 2.5 billion people lack improved sanitation [2]. Most of these people reside in rural settings where piped and other improved water supplies have failed to reach the dwellers [3], thus forcing rural communities to collect drinking water from untreated sources such as rivers and springs [4]. Unsafe drinking water, with poor sanitation and hygiene, accounts for nearly 10% of the total burden of disease worldwide. This includes an estimated 4 billion cases of diarrhea annually, causing 1.8 million deaths, mostly among children under 5 years of age [5].

South Africa is home to 51.8 million people [6] and approximately 40% of this population lives in small rural villages and scattered settlements [7]. Universal access to potable water and decent sanitation is a governmental objective in South Africa [8]; thus, the government has set a target of 100% coverage of potable water supply by 2014 [9]. Since 1994, access to potable water supply has improved significantly, from 59% of the population [10] to 95.2% in 2011 [11]. Still, around five million South Africans, mainly residing in rural areas, do not have a reliable source of safe drinking water [6], [11]. This situation exposes the rural communities to risks of acquiring water-borne diseases with resultant negative financial implications on the government when providing medical treatment to sick people, as these people predominantly use government clinics or hospitals for treatment [12]. For example, the South African government loses about $340 millionannually, due to approximately three million diarrhoeal cases and 50 000 related mortalities [13].

A systematic review by Wright et al. [14] showed that water contamination occurs between source and point-of-use. This pattern has been confirmed by subsequent studies of water contamination in rural Sierra Leone [15] and rural Honduras [16]. The extent of water contamination varies across the water supply chain (i.e., from source to cup used in a household), whereby more contamination is at the source than in a household setting [17].

Studies by Mpenyana-Monyani et al. [18] and Zamxaka et al. [19] reported concentrations of total coliform and *E. coli* which were higher than the limits set by WHO [20], SANS [21] and DWAF [22] for drinking water quality. As a result of the potential disease-causing characteristics of certain *E. coli*, the removal of *E. coli* from raw water is a major step in all drinking water purification systems [23].

Several studies have been done on the bacteriological water quality in some of the villages next to Tshaanda [17], [24]–[30]. The results of these studies showed that the levels of bacteriological concentration, which included *E. coli*, were above the recommended WHO and South African National Standards for drinking water quality and thus suggesting that the water was not fit for human consumption unless treated. Subsequently, ceramic filters [24], [31] were applied in an attempt to address water pollution at household level in the affected villages. Although this intervention significantly improved the quality of water and also reduced the incidence of diarrhoea, there was no follow up study to determine sustained use of filters [24]. The back drop of household water treatment options is that the responsibility of treating the water rests on the individual household. This could present variation in the quality of drinking water in the same village. For ceramic filters, tasks such as daily backwashing and cleaning of the filters could be considered tedious by some households and they may decide not to follow the instructions resulting in compromised quality of drinking water. Furthermore, some households may not have time to filter the water and would therefore drink untreated water. Monitoring of household water quality may be difficult to conduct due to restricted access to some households, for instance when there is no one in the house, thus increasing the duration of monitoring. On the other hand, population-based or source treatment is easy to monitor, can be accessed at any time and it saves time because the focus is on a single source. Furthermore, it provides uniform water quality at a given time thus becoming easy to determine which intervention should be implemented to improve water quality. Membrane filtration technology, particularly ultrafiltration (UF) system, can be used for source water treatment to provide potable water. The application of membrane filtration technology for potable water supply is well documented [32]–[40]. However, fouling is the most important factor that has limited the widespread use of membrane technology for the removal of microorganisms from drinking water. Feed pretreatment and membrane cleaning [41] are common strategies to minimize the fouling effect. Ultrafiltration systems have been used globally for treating various water resources including rivers, reservoirs, and lakes [42]–[44] for the production of potable water in the past decade. Ultrafiltration membranes with a pore size of between 0.1 and 0.01 micrometres provide an effective barrier for bacteria, viruses, suspended particles and colloids [45]–[47]. Ultrafiltration can be used on its own for treating drinking water where the feed water concentration is not too high in terms of organic contents [48]–[50]; for instance, if water turbidity is <100 NTU [51]. Accordingly, it has been used as pre-treatment for nanofiltration to reduce its fouling [50], [52] and for reverse osmosis (RO) to provide an excellent treated water quality [46], [47]. This has led to UF membranes replacing conventional pre-treatment procedures that consist of acid addition, coagulant/flocculant addition, chlorination, media filtration, and cartridge filtration [47].

Conventional large-scale ultrafiltration systems are operated at a transmembrane pressure of between 0.5–1.0 bar and require pumps for operation and backflushing. However, if operated by gravity, pump costs are avoided, and this can be an attractive option for decentralized, small-scale applications [45]. Presently, only few gravity-driven ultrafiltration systems for decentralized application exist (SkyJuice for community water supply and LifeStraw Family for household water treatment [51], [53]. These systems can be operated at ultra-low pressure (100–150 mbar) and require little maintenance compared to the conventionally operated UF [48]. Boulestreau et al. [51] successfully conducted trials at Ogunjini village in Kwa-Zulu Natal, South Africa with a decentralized, gravity-driven membrane system to treat river water and to produce 5 m^3^/d of drinking water. However, unlike our study, they used flat-sheet UF membranes. Furthermore, their system was not incorporated into any water supply infrastructure and treated water was not used for human consumption, instead it was conveyed back into the river.

Population-level potable water supply interventions such as UF, that are based on infrastructure network received less attention compared to point-of-use water treatment interventions which are being applied to improve water quality within homes and personal hygiene practices within households in rural areas of developing countries [3], [4], [14]–[17], [24], [54]. Furthermore, treatment of groundwater using decentralized gravity-fed ultrafiltration membrane technology for village-wide supply of potable water in South Africa's impoverished rural areas has not been tested. The aim of this study is to ascertain the use of ultrafiltration membrane for the provision of potable water in Tshaanda. The purpose of this article is to present the preliminary results of the ultrafiltration of groundwater for potable water supply in Tshaanda. The objectives of this study are to:

1. Demonstrate the feasibility of using membrane technology to provide potable water in a rural area;
2. Determine the bacteriological quality of raw water during low and high rainfall periods;
3. Determine the quality of the permeate against the WHO and South African national standards for drinking water quality.

## Methods

### Contextualization of Study Area

**Figure 1:**
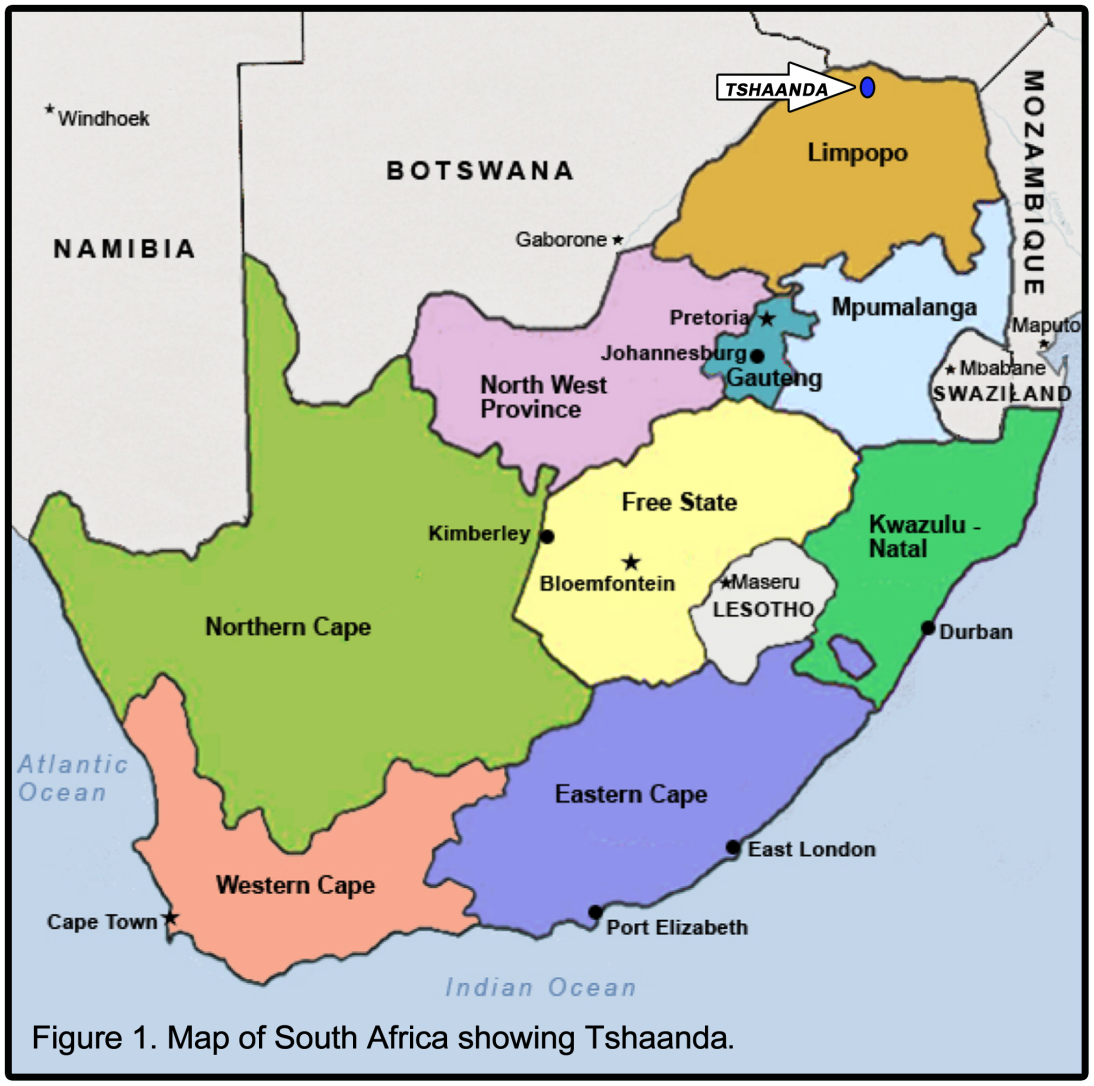
Map of South Africa showing Tshaanda.

Tshaanda village is situated in Vhembe District Municipality, in the Limpopo province, South Africa. The location of Tshaanda is shown in Figure 1. Tshaanda has approximately 800 people and 150 households. The profile of Tshaanda is presented in Table 1. The community gets water from an unprotected spring, which is the only source of water supply in the area. Tshaanda was chosen because it is one of the small villages that do not have safe drinking water infrastructure in Vhembe District Municipality jurisdiction. Furthermore, the village is impoverished and excluded from the mainstream water network in Vhembe and it is unlikely that the village will be connected to the mainstream water infrastructure in the future because the mountains that are surrounding it make Tshaanda difficult to access. The spring is situated deep in the mountain valleys and it can only be accessed by foot, thus posing a challenge, especially to older persons, people with disability, women, children and AIDS sufferers. The community shares this source with domestic animals. Furthermore, the community uses this source for bathing and washing clothes. Water supply infrastructure in Tshaanda is constituted of a diesel-driven engine, 60 000 L concrete water reservoir and five street taps.

**Table 1:** Tshaanda community profile [39].

Number of households	153
Population	835
Number of households with government subsidized houses	44
Number of households with Ventilated Improved Pit Toilets	63
Number of households without toilets	21
Source of water supply (spring)	1
Number of street water taps	8
Number of People with Matric (Grade 12)	50
Number of People with Tertiary qualification	12
Primary School	1
Clinic	0

No research study was done on water quality before in Tshaanda, therefore this study presented an opportunity to maximize the use of the water source and to also show case, based on the success of the project, the possibility of using membrane technology to provide safe drinking water in a rural area, especially since drinking water sources in rural areas are not monitored.

### Preliminary water sampling and analysis

On 18^th^ June 2012, three water samples were taken in Tshaanda in order to create baseline data of the quality of drinking water before treatment in order to compare it with the quality of water after treatment with UF membrane.

The quality of drinking water in Tshaanda was analysed in relation to the WHO [20] and South African [22] guidelines for drinking water quality. The water samples were analyzed for turbidity, pH, electrical conductivity, total coliform, enterococci and *E. coli* [55]. One water sample (500 ml) was taken at each of the three source points (*i.e.*, *Spring*, *Tap 1* and *Tap 2*). Distilled water (500 ml) which was obtained from the laboratory of the Department of Agriculture, Veterinary section in Makhado, Limpopo province, was used as a control.

Defined Substrate Technology was used for the bacteriological analysis of the water samples. The IDEXX Colilert was used to process the Total coliform and *E. coli* water samples, whereas IDEXX Enterolert was used to process enterococci samples in the laboratory. Water samples of IDEXX Colilert and IDEXX Enterolert were incubated for 22 hours at 37°C and 41°C, respectively. After incubation, total coliform was determined visually by identifying all test wells that turned yellow and subsequently, the Most Probable Number (MPN) was determined by counting the number of test wells that showed yellow colour. After determining the MPN, all the IDEXX Colilert Quanti-Tray plates were put individually under long wave (366 nm) ultraviolet light in order to detect any wells that had fluorescence [56]. The yellow wells that fluoresced were counted to determine the MPN for *E. coli*. A multi-meter (HACH) was used to measure the pH and electrical conductivity of the water samples. A portable turbidity meter (HACH) was used to measure the turbidity of the samples.

### Social and administrative processes (non-technical)

**Figure 2:**
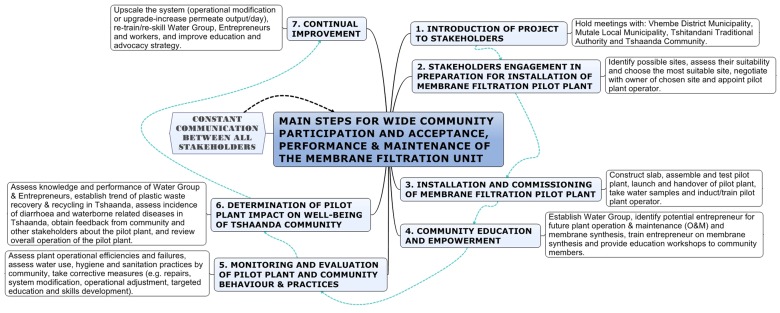
Main steps for wide community participation and acceptance, performance and maintenance of membrane filtration pilot plant.

Figure 2 highlights critical and fundamental issues that need to be adhered to in order to efficiently and successfully plan, implement and wind-up a water supply and management project in rural villages which, in most cases, are administered by traditional authorities in conjunction with local and/or district municipalities. Maintaining constant communication with the municipality, traditional authority and the community is very critical towards ensuring seamless implementation of the project and to promptly address concerns of all the key stakeholders. Furthermore, the water group will play a critical role of ensuring that the membrane pilot plant operates properly and that water related issues are resolved promptly and amicably. The water group will also ensure that the community takes ownership of the pilot plant, use it properly and protect it from vandalism and abuse. The group will educate the community about prevention of water pollution, responsible use of water and promotion of public health and personal hygiene.

### Ethics statement

The process started with seeking and obtaining approval to conduct the research in Tshaanda from Vhembe District Municipality, Mutale Local Municipality and Tshitandani Traditional Council, all of which have authority over the Tshaanda jurisdiction. The first meeting was held with the local chief of Tshitandani Traditional Authority in order to explain the purpose of the project to him and to obtain approval from the traditional authority to conduct the study in Tshaanda. Subsequent meetings were held with other key stakeholders (e.g., Vhembe District Municipality, Mutale Local Municipality and Tshaanda community) in order to present the research background, obtain approval and support from them and to discuss their roles during the entire duration of the research.

Written ethics approval for this research was obtained from Vhembe District Municipality, which has overall jurisdiction over Mutale Local Municipality, where Tshaanda is situated. Tshitandani Traditional Authority also gave a written ethics approval for the study to be conducted in Tshaanda, as it has sole traditional authority over the Tshaanda jurisdiction. The participants (whom were adults) from the community of Tshaanda gave their verbal consent to participate in this study during a community meeting, which was organized by the the local chief of Tshitandani Traditional Authority. They did so in the presence of the members of Tshitandani Traditional Authority. The community gave consent for instance, to participate in the discussions during community meetings and when establishing a local water group. Their consent was then recorded as part of the minutes of that meeting. It was sensible to ask for consent from the entire community (excluding any minor) in a meeting, because the study focuses on solving a community problem rather than an individual problem. Furthermore, the engagements with the participants were mainly through community meetings.

This study also received ethical approval from the institution review board committees at Tshwane University of Technology (TUT) and Katholieke Universiteit Leuven (KU Leuven).

### Public participation

A public participation process for the installation of a membrane filtration unit in Tshaanda was conducted by with the community of Tshaanda, Vhembe District Municipality, Mutale Local Municipality, Tshitandani Traditional Authority and Tshitandani Primary School Governing Body. Some of the key aspects that needed community involvement were the identification of a suitable location where the membrane filtration system should be installed, matters related to the security, operation and maintenance of the filtration system and the need for the establishment of local ‘Water Group’.

### Technical implementation

#### Raw water supply

Raw water was drawn from the local spring and pumped into a 2500 L raw water storage tank, which was placed at the top of the membrane filtration system. An existing diesel driven engine, which belongs to Vhembe District Municipality, was used to pump water from the spring to the raw water storage tank.

#### Membrane filtration system design, operation and maintenance

**Figure 3:**
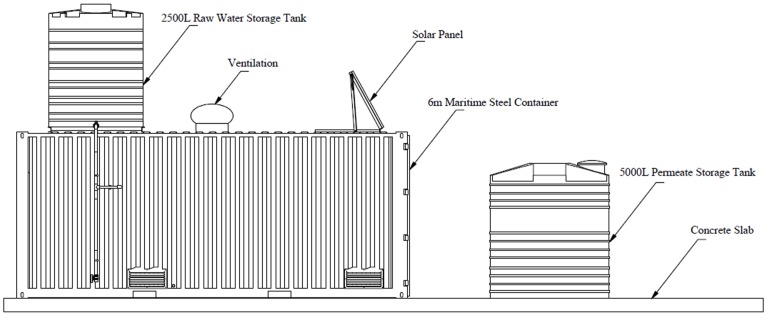
External design of the pilot plant in Tshaanda.

**Figure 4:**
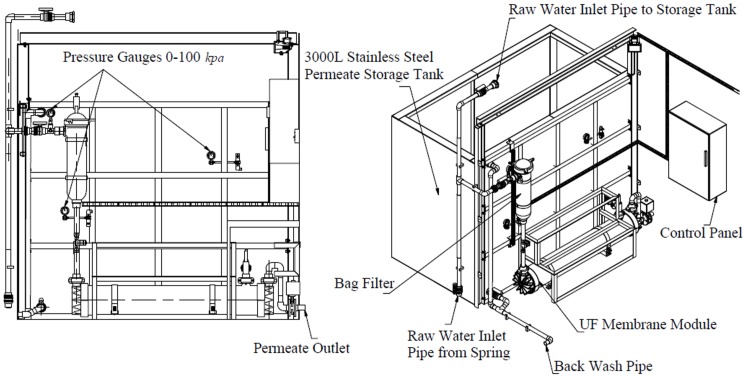
Internal design of the pilot plant in Tshaanda.

Gravity-driven ultrafiltration pilot plant was purchased from Ikusasa Water (A Division of Ikusasa Chemicals (Pty) Ltd and Chris Swartz Eng. (Water Utilization Engineers)) and it was installed in April 2013 at Tshaanda. The membrane filtration system was housed inside a 6 m maritime steel container. A hollow fibre UF membrane (Multibore membrane housed in a dizzer XL 1.5 MB 40 module), supplied by Inge watertechnologies AG, Germany), was used to drive the experiment in Tshaanda. The design of the pilot plant is shown in Figure 3 and Figure 4.

The plant was set to operate automatically even though it can be operated manually. The water flow rate was maintained between 300 and 400 L/h at a pressure of 2 kPa. The plant can produce 5 m^3^/day of potable water.

Due to the operational simplicity of the pilot plant, the operation and maintenance of the pilot plant was carried out by a member of the community, who was recommended by the community. He was then trained (1-day training) on how to operate and maintain the pilot plant by a technician from Ikusasa Water. Subsequent training of the plant operator was coupled with the main maintenance schedules. The plant maintenance included backflushing of the membrane module, cleaning of bagfilter, calibration of the control panel and correcting the flow rate. The main maintenance of the pilot plant was done once, every two months between April 2013 and February 2014 by Ikusasa Water, however; cleaning of the bagfilter was done once every week by the pilot plant operator. The first main maintenance was done in June 2013.

### Water sampling and analysis

Three months after the pilot plant was installed, water samples of raw (untreated) and permeate (treated) were taken for analysis on the 25^th^ July 2013 in order to determine whether the pilot plant was able to produce water that met the requirements of the WHO and South African water quality guidelines [20], [22] and standards [21]. Two sets of raw water samples (one from the spring, labelled as ***Spring*** and another sample at the inlet of the membrane filtration plant just before the bagfilter, labelled as ***Plant inlet before filtration***) and four samples of treated water were taken (one from the storage tank inside the filtration plant, labelled as ***Permeate*** and three from the three taps outside the plant, labelled as ***Tap 1***, ***Tap 2*** and ***Tap 3***, respectively). Furthermore, other samples were taken on the 25^th^ February 2014 in order to determine the quality of water during wet season. The water samples were taken aseptically and sterile zip-lock water bags were used. The water samples were put inside a cooler bag with ice to maintain the temperature at ∼4°C, until they were processed for analysis in the laboratory. The distance between the area where the samples were taken and the laboratory where they were analysed was 160 km. Accordingly, the samples were processed within 6 hours after collection. The samples were processed according to the same procedure that was used during the preliminary investigation of the water quality.

## Results and Discussion

### Installation and operation of pilot plant

The pilot membrane filtration plant was installed on the premises of the local primary school following an agreement between the community and the school governing body. The pilot plant was installed approximately 600 m away from the water source (*i.e.*, spring), construction and commissioning phases were completed within one month. The system operated through gravity for 24 h/day and produced 5000 L of potable water per day for the community of Tshaanda.

### Water quality analysis

The water samples were processed and analyzed for microbiological and physico-chemical parameters. Microbiological analysis was carried out in order to determine the presence of *E. coli*, enterococci and total coliform in the water samples. Physico-chemical analysis was carried out to determine the levels of pH, turbidity and electrical conductivity, respectively.

The results of the preliminary water quality investigation are presented in Table 2.

**Table 2:** Results of the preliminary water samples taken in Tshaanda on 18 June 2012.

SOURCE	CHEMICAL	GUIDELINES *(DWAF 1996 & WHO 2011)*	BACTERIOLOGICAL *(Indicator of fecal pollution)*	MPN	GUIDELINES *(DWAF 1996 & WHO 2011)*
**SPRING**	pH = 7.78	5 to 8	Total Coliform = (29/4)	35	<10 cfu/100 ml
	Conductivity = 46.7 µs/cm		*E. Coli* = (15/2)	17.4	0 cfu/100 ml
	Turbidity = 0.52	1 to 5 NTU	Enterococci = (7)	7	0 cfu/100 ml
**TAP 1 ** ***(street tap)***	pH = 7.38	5 to 8	Total Coliform = (26/2)	29	<10 cfu/100 ml
	Conductivity = 43.1 µs/cm		*E. Coli* = (16)	16.1	0 cfu/100 ml
	Turbidity = 0.51	1 to 5 NTU	Enterococci = (0/0)	<1	0 cfu/100 ml
**TAP 2 ** ***(street tap)***	pH = 7.39	5 to 8	Total Coliform = (15)	15.1	<10 cfu/100 ml
	Conductivity = 40.8 µs/cm		*E. Coli* = (3)	3	0 cfu/100 ml
	Turbidity = 4.31	1 to 5 NTU	Enterococci = (0)	<1	0 cfu/100 ml
**CONTROL**			Total Coliform = (0)	<1	0 cfu/100 ml
			*E. Coli* = (0)	<1	0 cfu/100 ml
			Enterococci = (0)	<1	0 cfu/100 ml

Note: **DWAF**-Formerly, Department of Water Affairs & Forestry, now Department of Water Affairs (DWA). **MPN**-Most Probable Number.

Critical analysis of Table 2 shows that turbidity values of all the samples were within the WHO and South African standards limits for drinking water quality. The results also show the presence of *E. coli*, enterococci and total coliform in concentrations above the recommended limits.

The results of the raw water and treated water samples are shown in Table 3 and Table 4, respectively.

**Table 3:** Results of the water samples taken in Tshaanda on 25 July 2013.

SOURCE	CHEMICAL	GUIDELINES *(DWAF 1996 & WHO 2011)*	BACTERIOLOGICAL *(Indicator of fecal pollution)*	MPN	GUIDELINES *(DWAF 1996 & WHO 2011)*
**SPRING**	pH = 6.45	5 to 8	Total Coliform = (46)	118.4	<10 cfu/100 ml
	Conductivity = 49.3 µs/cm		*E. Coli* = (6)	6.4	0 cfu/100 ml
	Turbidity = 1.01	1 to 5 NTU	Enterococci = (3)	3.1	0 cfu/100 ml
**PLANT INLET (Before filtration)**	pH = 6.31	5 to 8	Total Coliform = (21)	27.1	<10 cfu/100 ml
	Conductivity = 47.1 µs/cm		*E. Coli* = (0)	<1.0	0 cfu/100 ml
	Turbidity = 1.69	1 to 5 NTU	Enterococci = (1)	1.0	0 cfu/100 ml
**PERMEATE**	pH = 6.14	5 to 8	Total Coliform = (50)	200.5	<10 cfu/100 ml
	Conductivity = 53.6 µs/cm		*E. Coli* = (0)	<1	0 cfu/100 ml
	Turbidity = 0.61	1 to 5 NTU	Enterococci = (0)	<1	0 cfu/100 ml
**TAP 1**	pH = 6.10	5 to 8	Total Coliform = (50)	200.5	<10 cfu/100 ml
	Conductivity = 48.5 µs/cm		*E. Coli* = (0)	<1	0 cfu/100 ml
	Turbidity = 2.09	1 to 5 NTU	Enterococci = (0)	<1	0 cfu/100 ml
**TAP 2**	pH = 6.11	5 to 8	Total Coliform = (50)	200.5	<10 cfu/100 ml
	Conductivity = 48.1 µs/cm		E. Coli = (0)	<1	0 cfu/100 ml
	Turbidity = 1.34	1 to 5 NTU	Enterococci = (0)	<1	0 cfu/100 ml
**TAP 3**	pH = 6.08	5 to 8	Total Coliform = (50)	200.5	<10 cfu/100 ml
	Conductivity = 48.2 µs/cm		E. Coli = (0)	<1	0 cfu/100 ml
	Turbidity = 0.88	1 to 5 NTU	Enterococci = (0)	<1	0 cfu/100 ml
**CONTROL**			Total Coliform = (0)	<1	0 cfu/100 ml
			E. Coli = (0)	<1	0 cfu/100 ml
			Enterococci = (0)	<1	0 cfu/100 ml

Note: **DWAF**-Formerly, Department of Water Affairs & Forestry, now Department of Water Affairs (DWA). **MPN**-Most Probable Number.

**Table 4:** Results of the water samples taken in Tshaanda on 25 February 2014.

SOURCE	CHEMICAL	GUIDELINES *(DWAF 1996 & WHO 2011)*	BACTERIOLOGICAL *(Indicator of fecal pollution)*	MPN	GUIDELINES *(DWAF 1996 & WHO 2011)*
**SPRING**	pH = 7.04	5 to 8	Total Coliform = (49/48)	>2419.2	<10 cfu/100 ml
	Conductivity = 45.4 µs/cm		*E. Coli* = (27/2)	40.4	0 cfu/100 ml
	Turbidity = 0.82	1 to 5 NTU	Enterococci = (37/5)	73.3	0 cfu/100 ml
**PLANT INLET (Before filtration)**	pH = 7.17	5 to 8	Total Coliform = (49/10)	204.6	<10 cfu/100 ml
	Conductivity = 46.8 µs/cm		*E. Coli* = (24/0)	31.7	0 cfu/100 ml
	Turbidity = 0.72	1 to 5 NTU	Enterococci = (7/1)	8.5	0 cfu/100 ml
**PERMEATE**	pH = 6.93	5 to 8	Total Coliform = (15)	15.1	<10 cfu/100 ml
	Conductivity = 51.9 µs/cm		*E. Coli* = (0)	<1	0 cfu/100 ml
	Turbidity = 0.46	1 to 5 NTU	Enterococci = (0)	<1	0 cfu/100 ml
**CONTROL**			Total Coliform = (0)	<1	0 cfu/100 ml
			*E. Coli* = (0)	<1	0 cfu/100 ml
			Enterococci = (0)	<1	0 cfu/100 ml

Note: **DWAF**-Formerly, Department of Water Affairs & Forestry, now Department of Water Affairs (DWA). **MPN**-Most Probable Number.

### Total Coliform

Detection of total coliform in water indicates potential faecal pollution and thus provide information on treatment efficiency and after growth [21]. After a period of more than 20 months (June 2012–February 2014), total coliform counts of raw water was above the acceptable SANS limit. Furthermore, there was a significant increase in the total coliform count in February 2014 (>2419.2 cfu/100 ml) due to heavy rains that were experienced in the area two weeks before sampling. In July 2013, total coliform of the permeate was about 4 times (200.5 cfu/100 ml) more than that of the untreated water source (48 cfu/100 ml). This was because the permeate tank was not covered on top and therefore it presented an easy passage of dust into the permeate. The tank was subsequently cleaned and covered with a canvas. In February 2014, the coliform count of the permeate was 7.4 cfu/100 ml, following the cleaning of the permeate tank, thus being within the acceptable limit.

### *Escherichia coli* and enterococci

*Escherichia coli* is the best indicator microorganism of faecal pollution [57]. The overall quality of the water at the spring was found to be consistently unsafe for drinking purposes due to the presence of *E.coli* in the water samples taken. After a period of more than 20 months (June 2012–February 2014), *E. coli* in raw water increased by more than 2 times (17.4 cfu/100 ml in June 2012 as against 40.4 cfu/100 ml in February 2014). The number of enterococci at the water source increased ten folds between June 2012 and February 2014 (7 cfu/100 ml in June 2012 and 73.3 cfu/100 ml) due to rain. This was expected as increased levels of microbial pollution are usually experienced during rainy seasons, since large numbers of microorganisms are washed from various point- and non-point pollution sites [58]. However, the concentration of enterococci was significantly lower at the filtration unit (Plant inlet before filtration). This could probably be attributed to die-off as bacteria compete for limited oxygen and nutrients in the water [59].

This water poses a public health risk to the community of Tshaanda if consumed untreated and could lead to waterborne diseases, such as: hepatitis A and E, cholera, typhoid and poliomyelitis [3]. Consuming water contaminated with *E. coli* produce a 15% chance of of having diarrhea.

Following ultrafiltration, *E. coli* and enterococci were undetected (<1 cfu/100 ml) in the permeate samples taken in July 2013 and February 2014 respectively. Maintaining this water quality and ensuring that potable water is available in sufficient quantity will help to reduce the diarrhea episodes [12], [60]–[62].

### Turbidity

The turbidity values recorded for all the water samples were within the WHO [20] and SANS [21] acceptable limits for drinking water (<5 NTU) during dry and rainy seasons (June 2012, July 2013 and February 2014). Interestingly, the turbidity of the permeate was almost two times lower than that of the spring between July 2013 and February 2014. These observed lower turbidity values suggest that there is no need for pre-treatment of the water for suspended particles and colloids [51].

### pH and electrical conductivity

The pH and electrical conductivity levels of all the water samples were within the recommended limits of WHO [20] and SANS [21] for drinking water quality.

### Challenges of the project

The main challenge encountered was that there was no formal procedure for ordering and supplying diesel which at times delayed the supply of the diesel to Tshaanda. This affected pumping of raw water to the pilot plant, consequently leading to operational stoppage of the pilot plant. In order to address these challenges, a plan was developed, whereby Vhembe District Municipality supplied 220 L of diesel on a monthly basis. Furthermore, the plant operator would notify the local councillor when it was time to order diesel, the local councillor would request the responsible manager at Vhembe District Municipality to deliver the diesel to Tshaanda. The plan also included an institutional arrangement whereby other parties (i.e., KU Leuven or Tshwane University of Technology) would provide assistance to repair the engine or the water pump in case Vhembe District Municipality needed support.

### Conclusions

A gravity driven ultrafiltration membrane pilot plant was used for ten (10) months (from April 2013 to February 2014) to produce potable water to the rural community of Tshaanda. The preliminary results showed that the microbiological quality of the permeate was within recommended and acceptable limits of the WHO and South African standards for drinking water quality.

Following the successful removal of *E. coli*, it can be inferred that the membrane system in Tshaanda was able to produce safe drinking water. This further confirms that the integrity of the filtration membrane was still maintained and that it was still highly effective in removing indicator microorganisms, suspended solids and colloidal matter [63] after ten (10) months of operation.

Though the UF pilot plant in Tshaanda could produce safe drinking water, there is still much work to be done before this technology could be rolled-out country wide. Subsequent research will be expanded to include aspects of evidence of sustained use, positive health impact, water quality over extended periods of use, operation and maintenance, cost-effectiveness, system performance and optimization (considering seasonal variations), and water quality monitoring, whereby the frequency of water testing will be increased. Furthermore, future research will concentrate on the water distribution network to determine bacterial regrowth and water quality deterioration, public perception, household health behaviors, acceptability of the technology, community outreach and awareness raising on water, sanitation and hygiene. Acquiring this information would help in ensuring that the system continues to deliver safe water in the long run.

## References

[1] World Health Organization and UNICEF (2013) Progress on Drinking Water and Sanitation: 2013 Update. Available: http://www.wssinfo.org/fileadmin/user_upload/resources/JMPreport2013.pdf. Accessed: 2013 September 07.

[2] World Health Organization, UNICEF (2012) Progress on Drinking Water and Sanitation: 2012 Update. Available: http://www.unicef.org/media/files/JMPreport2012.pdf. Accessed: 2013 May 24.

[3] Rosa G, Clasen T (2010) Estimating the scope of household water treatment in low-and medium-income countries. The American Journal of Tropical Medicine and Hygiene 82: 289–300. 20134007 10.4269/ajtmh.2010.09-0382 PMC2813171

[4] Sobsey MD, Water S (2002) Managing water in the home: accelerated health gains from improved water supply. Geneva: World Health Organization.

[5] World Health Organization (2005) The World Health Report 2005: Make every mother and child count. Geneva: World Health Organization.

[6] Statistics South Africa (2012) Census 2011 Statistical release – P0301.4/Statistics South Africa. Pretoria. Available: http://www.statssa.gov.za/Publications/P03014/P030142011.pdf. Accessed: 2013 May 24.

[7] de la Harpe J (2011) South Africa: Lessons for Rural Water Supply; Assessing progress towards sustainable service delivery. The Hague: IRC International Water and Sanitation Centre. Available: http://www.washinschools.info/docsearch/title/179989. Accessed: 2013 April 30.

[8] Republic of South Africa (1996) Constitution of the Republic of South Africa Act, 1996 (Act No. 108 of 1996). Pretoria: Government Printing Works.

[9] Department of Water Affairs (DWA) (2012) Briefing of the Portfolio Committee: Water & Environmental Affairs: Progress and Perspectives on Water Supply Millennium Development Goals. Available: http://www.pmg.org.za/report/20121018-department-water-affairs-attainment-millennium-development-goals. Accessed: 2013 May 24.

[10] Wall K (2011) SAICE infrastructure report card for South Africa 2011. SAICE. Available: http://www.csir.co.za/enews/2011_jun/download/infrastructure_report_card_sa_2011.pdf. Accessed: 2013 May 24.

[11] Department of Water Affairs (2013) Speech by the Minister of Water and Environmental Affairs, Mrs BEE Molewa, on the occasion of the presentation of Budget Vote Number 37 on behalf of the Department of Water Affairs at the Old Assembly Chamber, Parliament, Cape Town, 21 May 2013. Available: http://www.dwa.gov.za/Communications/PressReleases/2013/STATEMENT%20BY%20MINISTER%20EDNA%20MOLEWA%20-BUDGET%20VOTE%20%20MEDIA%20BRIEFING%202013%20L%20PA.pdf. Accessed: 2013 May 24.

[12] Cameron J, Jagals P, Hunter PR, Pedley S, Pond K (2011) Economic assessments of small-scale drinking-water interventions in pursuit of MDG target 7C. Science of the Total Environment 410: 8–15. 22014511 10.1016/j.scitotenv.2011.09.054

[13] Momba M, Obi C, Thompson P (2009) Survey of disinfection efficiency of small drinking water treatment plants: Challenges facing small water treatment plants in South Africa. Water SA 35: 485–493.

[14] Wright J, Gundry S, Conroy R (2004) Household drinking water in developing countries: a systematic review of microbiological contamination between source and point-of-use. Tropical Medicine & International Health 9: 106–117. 14728614 10.1046/j.1365-3156.2003.01160.x

[15] Clasen T, Bastable A (2003) Faecal contamination of drinking water during collection and household storage: the need to extend protection to the point of use. J Water Health 1: 109–115. 15384721

[16] Trevett A, Carter R, Tyrrel S (2005) The importance of domestic water quality management in the context of faecal-oral disease transmission. J Water Health 3: 259–270. 16209030 10.2166/wh.2005.037

[17] Gundry S, Wright J, Conroy R, Du Preez M, Genthe B (2013) Contamination of drinking water between source and point-of-use in rural households of South Africa and Zimbabwe: implications for monitoring the Millennium Development Goal for water. Water Practice and Technology 1: 1–9.

[18] Mpenyana-Monyatsi L, Onyango MS, Benteke Momba MN (2012) Groundwater Quality in a South African Rural Community: A Possible Threat to Public Health. Polish Journal of Environmental Studies 21: 1349–1358.

[19] Zamxaka M, Pironcheva G, Muyima N (2004) Microbiological and physico-chemical assessment of the quality of domestic water sources in selected rural communities of the Eastern Cape Province, South Africa. Water SA 30: 333–340.

[20] World Health Organization (2011) Guidelines for Drinking Water Quality. Available: http://whqlibdoc.who.int/publications/2011/9789241548151_eng.pdf. Accessed 2013 October 15.

[21] South African Bureau of Standards (2011) Drinking Water - SANS 241-1: 2011. Part 1: Microbiological, physical, aesthetic and chemical determinands. Pretoria: SABS Standards Division.

[22] Department of Water Affairs and Forestry (DWAF) (1996) South African Water Quality Guidelines Volume 1: Domestic Water Use Second Edition, Pretoria. Available: http://www.dwaf.gov.za/IWQS/wq_guide/domestic.pdf. Accessed: 2013 April 22.

[23] Varkey A, Dlamini M (2012) Point-of-use water purification using clay pot water filters and copper mesh. Water SA 38: 721–726.

[24] du Preez M, Conroy RM, Wright JA, Moyo S, Potgieter N, et al (2008) Short report: use of ceramic water filtration in the prevention of diarrheal disease: a randomized controlled trial in rural South Africa and Zimbabwe. American Journal of Tropical Medicine and Hygiene 79: 696–701. 18981506

[25] Potgieter N, Obi CL, Bessong PO, Igumbor EO, Samie A, et al (2005) Bacteriological contamination of vhuswa, a local weaning food, and stored drinking water in impoverished households in the Venda region of South Africa. J Health, Pop Nutr 23: 150–155. 16117367

[26] Potgieter N, Mudau LS, Maluleke FRS (2006) The microbiological quality of ground water sources used by rural communities in the Limpopo Province, South Africa. Water, Science and Technology 54 (11) 371–377. 10.2166/wst.2006.890 17302341

[27] Wright J, Gundry SW, Conroy R, Du Preez M, Genthe B, et al (2009) Child dysentery in the Limpopo Valley: a cohort study of water, sanitation, and hygiene risk factors. Journal of Water and Health 7: 259–266. 19240352 10.2166/wh.2009.032

[28] Obi CL, Potgieter N, Bessong PO, Matsaung G (2002) Assessment of the microbiological quality of river water sources in rural Venda communities in South Africa. Water SA 28: 287–291. 12639006

[29] Obi CL, Potgieter N, Bessong PO, Matsaung G (2003) Scope of potential bacterial agents of diarrhoea and microbial assessment of quality of river water sources in rural Venda communities in South Africa. Wat Sci Tech 47: 59–64. 12639006

[30] Du Preez M, le Roux W, Potgieter N, Venter SN (2008) The genetic relatedness of E. coli associated with post-collection drinking water contamination in rural households. Water SA 34: 107–111.

[31] Potgieter N, Becker PJ, Ehlers MM (2009) Evaluation of the CDC safe water-storage intervention to improve the microbiological quality of point-of-use drinking water in rural communities in South Africa. Water SA 35: 505–516.

[32] Watson BM, Hornburg CD (1989) Low-Energy Membrane Nanofiltration for Removal of Color, Organics and Hardness from Drinking-Water Supplies. Desalination 72: 11–22.

[33] Cartwright P (1991) Zero discharge/water reuse—The opportunities for membrane technologies in pollution control. Desalination 83: 225–241.

[34] Rachwal A, Khow J, Colbourne J, O'Donnell J (1994) Water treatment for public supply in the 1990's—A role for membrane technology? Desalination 97: 427–436.

[35] Kunikane S, Magara Y, Itoh M, Tanaka O (1995) A comparative study on the application of membrane technology to the public water supply. Journal of membrane science 102: 149–154.

[36] Ventresque C, Bablon G (1997) The integrated nanofiltration system of the Mery-sur-Oise surface water treatment plant (37 mgd). Desalination 113: 263–266.

[37] Arnal JMA, Fernandez MS, Martin GV, Garcia JL, Zafrilla JMG, et al (2002) Design and construction of a water potabilization membrane facility and its application to the third world countries. Preliminary tests. Desalination 145: 305–308.

[38] Van der Bruggen B, Vandecasteele C (2003) Removal of pollutants from surface water and groundwater by nanofiltration: overview of possible applications in the drinking water industry. Environmental Pollution 122: 435–445. 12547533 10.1016/s0269-7491(02)00308-1

[39] Muhammad N, Sinha R, Krishnan ER, Patterson CL (2009) Ceramic filter for small system drinking water treatment: Evaluation of membrane pore Size and importance of integrity monitoring. Journal of Environmental Engineering 135: 1181–1191.

[40] Fane A, Tang C, Wang R (2011) Membrane technology for water: microfiltration, ultrafiltration, nanofiltration, and reverse osmosis. Treatise on Water Science, Academic Press, Oxford 4: 301–335.

[41] Pangarkar BL, Sane MG, Guddad M (2011) Reverse osmosis and membrane distillation for desalination of groundwater: A review. ISRN Materials Science. http://dx.doi.org/10.5402/2011/523124.

[42] Laîné JM, Vial D, Moulart P (2000) Status after 10 years of operation—overview of UF technology today. Desalination 131: 17–25.

[43] Murthy Z, Chaudhari L (2009) Treatment of distillery spent wash by combined UF and RO processes. Global NEST Journal 11: 235–240.

[44] Manjunath D, Madhumala M, Prasad R, Kalyani S, Sridhar S (2013) Processing of surface and ground water by hydrostatic pressure-driven membrane techniques: design and economic aspects. Desalination and Water Treatment 51: 5873–5885.

[45] Peter-Varbanets M, Johnston R, Meierhofer R, Kage F, Müller S, et al.. (2011) Gravity Driven Membrane Disinfection for household drinking water treatment. 35th WEDC International Conference, Loughborough, UK.

[46] Pearce G (2007) The case for UF/MF pretreatment to RO in seawater applications. Desalination 203: 286–295.

[47] Fernández G, Plaza F, Garralón G, Garralón A, Pérez JI, et al (2012) A comparative study of ultrafiltration and physicochemical process as pretreatment of seawater reverse osmosis. Desalination and Water Treatment 42: 73–79.

[48] Peter-Varbanets M, Zurbrügg C, Swartz C, Pronk W (2009) Decentralized systems for potable water and the potential of membrane technology. Water Research 43: 245–265. 19010511 10.1016/j.watres.2008.10.030

[49] Yu Z, Chu H, Cao D, Ma Y, Dong B, et al (2012) Pilot-scale hybrid bio-diatomite/dynamic membrane reactor for slightly polluted raw water purification. Desalination 285: 73–82.

[50] Zheng X, Yu M, Liang H, Qi L, Zheng H, et al (2012) Membrane technology for municipal drinking water plants in China: progress and prospect. Desalination and Water Treatment 49: 281–295.

[51] Boulestreau M, Hoa E, Peter-Verbanets M, Pronk W, Rajagopaul R, et al (2012) Operation of gravity-driven ultrafiltration prototype for decentralised water supply. Desalination and water treatment 42: 125–130.

[52] Ferella F, De Michelis I, Zerbini C, Veglio F (2013) Advanced treatment of industrial wastewater by membrane filtration and ozonization. Desalination 313: 1–11.

[53] Clasen T, Naranjo J, Frauchiger D, Gerba C (2009) Laboratory assessment of a gravity-fed ultrafiltration water treatment device designed for household use in low-income settings. The American Journal of Tropical Medicine and Hygiene 80: 819–823. 19407130

[54] Brown J, Proum S, Sobsey M (2008) Escherichia coli in household drinking water and diarrheal disease risk: evidence from Cambodia. Water Science & Technology 58: 757–763. 18776609 10.2166/wst.2008.439

[55] World Health Organization (2008) Guidelines for Drinking-water Quality. Available: http://www.who.int/water_sanitation_health/dwq/fulltext.pdf. Accessed: 2013 September 23.

[56] Landre J, Gavriel A, Lamb A (1998) False-positive coliform reaction mediated by Aeromonas in the Colilert defined substrate technology system. Letters in applied microbiology 26: 352–354. 9674164 10.1046/j.1472-765x.1998.00347.x

[57] Edberg S, Rice E, Karlin R, Allen M (2000) Escherichia coli: the best biological drinking water indicator for public health protection. Journal of Applied Microbiology 88: 106S–116S. 10.1111/j.1365-2672.2000.tb05338.x 10880185

[58] Dobrowksy P, van Deventer A, De Kwaadsteniet M, Ndlovu T, Khan S, et al (2013) Prevalence of virulence genes associated with pathogenic Escherichia coli strains isolated from domestically harvested rainwater during low and high rainfall periods. Appl Environ Microbiol doi:10.1128/AEM.03061-13 10.1128/AEM.03061-13 PMC3957612 24375127

[59] Momba MN, Notshe T (2003) The microbiological quality of groundwater-derived drinking water after long storage in household containers in a rural community of South Africa. Journal of Water Supply: Research & Technology-AQUA 52: 67–77.

[60] Cameron J, Hunter P, Jagals P, Pond K (2011) Valuing water, valuing livelihoods. London: IWA Publishing.

[61] Esrey SA, Habicht J (1985) The impact of improved water supplies and excreta disposal facilities on diarrheal morbidity, growth, and mortality among children. Cornell International Nutrition Monograph Series 15: 1–85.

[62] Esrey SA, Feachem RG, Hughes JM (1985) Interventions for the control of diarrhoeal diseases among young children: improving water supplies and excreta disposal facilities. Bulletin of the World Health organization 63: 757–772. 3878742 PMC2536385

[63] Hofman J, Beumer M, Baars E, Van der Hoek J, Koppers H (1998) Enhanced surface water treatment by ultrafiltration. Desalination 119: 113–125.

